# Ion-Selective Electrodes with Solid Contact Based on Composite Materials: A Review

**DOI:** 10.3390/s23135839

**Published:** 2023-06-23

**Authors:** Cecylia Wardak, Karolina Pietrzak, Klaudia Morawska, Malgorzata Grabarczyk

**Affiliations:** 1 Department of Analytical Chemistry, Institute of Chemical Sciences, Faculty of Chemistry, Maria Curie-Sklodowska University, Maria Curie-Sklodowska Square. 3, 20-031 Lublin, Poland; klaudiamorawska0905@gmail.com (K.M.); malgorzata.grabarczyk@mail.umcs.pl (M.G.); 2 Department of Food and Nutrition, Medical University of Lublin, 4a Chodzki Str., 20-093 Lublin, Poland; karolina.pietrzak@umlub.pl

**Keywords:** ion-selective electrodes, potentiometry, solid contact, composite, nanocomposite

## Abstract

Potentiometric sensors are the largest and most commonly used group of electrochemical sensors. Among them, ion-selective electrodes hold a prominent place. Since the end of the last century, their re-development has been observed, which is a consequence of the introduction of solid contact constructions, i.e., electrodes without an internal electrolyte solution. Research carried out in the field of potentiometric sensors primarily focuses on developing new variants of solid contact in order to obtain devices with better analytical parameters, and at the same time cheaper and easier to use, which has been made possible thanks to the achievements of material engineering. This paper presents an overview of new materials used as a solid contact in ion-selective electrodes over the past several years. These are primarily composite and hybrid materials that are a combination of carbon nanomaterials and polymers, as well as those obtained from carbon and polymer nanomaterials in combination with others, such as metal nanoparticles, metal oxides, ionic liquids and many others. Composite materials often have better mechanical, thermal, electrical, optical and chemical properties than the original components. With regard to their use in the construction of ion-selective electrodes, it is particularly important to increase the capacitance and surface area of the material, which makes them more effective in the process of charge transfer between the polymer membrane and the substrate material. This allows to obtain sensors with better analytical and operational parameters. Brief characteristics of electrodes with solid contact, their advantages and disadvantages, as well as research methods used to assess their parameters and analytical usefulness were presented. The work was divided into chapters according to the type of composite material, while the data in the table were arranged according to the type of ion. Selected basic analytical parameters of the obtained electrodes have been collected and summarized in order to better illustrate and compare the achievements that have been described till now in this field of analytical chemistry, which is potentiometry. This comprehensive review is a compendium of knowledge in the research area of functional composite materials and state-of-the-art SC-ISE construction technologies.

## 1. Introduction

Along with the development of technology and increasing processing of raw materials used in various industries, there is a growing demand for research aimed at monitoring the ongoing changes and the general condition of the natural environment. They are caused by both natural processes over which humans have no direct influence and by the activities of society. Devices that allow to quickly and easily determine the content of ions in various types of environmental samples are ion-selective electrodes (ISEs), which are currently the most popular potentiometric sensors and have been the subject of research for many scientists around the world continuously for many years, thanks to their numerous advantages [1,2]. The enormity of possibilities offered by modifications of ion-selective electrodes, including the development of wearable sensors [3,4] and multi-sensor platforms [5,6,7,8] for simultaneous monitoring of ion concentrations in a continuous mode in the in situ environment, causes that research on new constructions of electrodes to achieve better and better analytical and electrical parameters is still being published [9]. ISEs are currently used to determine the concentration of various types of ions (both inorganic and organic) in liquid samples in many areas of human life (agriculture [10,11], food industry [11,12,13] and pharmaceutical industry [14,15], process control or clinical diagnostics [16,17,18,19]). ISEs can be used to determine the content of selected ions in natural water samples, both surface waters (rivers, lakes and seas) and groundwater, as well as in tap water and sewage [20,21,22,23].

There are many review articles available in the scientific literature focusing on the collection and comparison of groups of substances and/or materials, their properties (including mechanical and electrical) and their applications in various industries. Nanocomposites consisting of polymers and carbon nanomaterials [24,25,26], as well as nanocomposites of polymers and ionic liquids [27], are widely described. The variety of composite materials and the number of possibilities of synthesis and modifying their composition or/and type of components can be a reason for their great popularity among scientists. Depending on the desired properties of materials and their future applications, it is possible to obtain composites that will be characterized by similar parameters, or most often even much better than the parameters of their individual components. Many review articles focus mainly on the method of synthesis of new materials and their parameters measured by multiple research methods. This article focuses on the use of nanocomposite materials in the construction of solid contact electrodes, in which they act as an ion–electron transducer (or sometimes even as an active component) and allow to obtain sensors with very good electrical and analytical parameters. Taking into account the type of materials used in the synthesis of nanocomposites, they can be divided into three main groups: composite materials obtained from conductive polymers and carbon nanomaterials; materials based on carbon nanomaterials and conductive polymers in combination with other materials, i.e., nanoparticles of metals and metal oxides, ionic liquids, molecular organic materials or zeolites. Selected analytical parameters of the electrodes described in the literature are summarized in the form of a table, which enables a quick comparison between the types of electrodes constructed in recent years, depending on the ions they determine and their capabilities.

## 2. Potentiometry and Ion-Selective Electrodes

Potentiometry is a highly selective and relatively cheap method that allows achieving low detection limits and a very wide dynamic range of sensors (up to eight orders of units) [28,29]. The principle of the method is to measure the electromotive force (EMF) of a cell made of two types of electrodes: a reference electrode whose potential has to necessarily be constant regardless of the composition and concentration of the sample, and an indicator (working) electrode whose potential changes depending on the activity of the main ion present in the sample solution to which the ion-selective membrane is sensitive [30]. A potentiometric response is obtained, which is dependent on potential changes in real time [31]. In most cases, no steps are necessary to prepare the sample for measurement. Moreover, the wide range of linearity of the method allows to avoid dilution or concentration of the sample solution, as is often necessary using ASA or HPLC methods. Most often (if necessary), it is limited to a small addition of ionic strength buffer and/or other substances to the sample solution, immediately before measurement and mixing. Sometimes it can also be necessary to ensure the correct pH of the environment when the pH of the test sample is not in the range, where the electrodes can work without interference. However, in most cases, this range is wide enough to allow the determination of ions directly in the collected sample. The great advantage of ISEs is the ability to test colored or muddy solutions, because both the color and the presence of solid particles do not interfere with the tests.

Among the various constructions of ISEs, a special place is held by electrodes without an internal electrolyte solution, the so-called ion-selective electrodes with solid contact (SCISEs). SCISEs are much more convenient during use, transport and storage, and they are easier to miniaturize and modify their shape and also more mechanically resistant. In addition, they can work in any position and in conditions of increased pressure and temperature, which results from the elimination of the internal solution present in classic electrodes, which acts as a link between the discharge electrode and the ion-selective membrane [13]. For electrodes of this type, it is very important to properly select the material that will function as an ion–electron transducer, thus enabling the correct operation of the electrodes by ensuring appropriate stability and reproducibility of the potential. When developing a new type of SCISEs, there are two main ways to improve their analytical parameters. The first is the use of a new active substance which as a membrane component is responsible for the appropriate selectivity of the sensors towards the selected main ion (especially important in the study of complex samples containing interferences that may interfere with the proper measurement), and the second is the search for new electroactive materials that can be successfully used as solid contact. The use of solid contact is additionally aimed at obtaining a satisfactory stability of the sensor potential, which will allow doing measurements for a longer period of time (weeks, months), often without the need for frequent calibration [9]. Electrodes used for this purpose should also be resistant to changes in measurement conditions (temperature, lighting and the presence of gases) [32]. Due to the common trend towards miniaturization of devices (also due to much smaller amounts of materials used for their construction and compatibility with small volumes of samples necessary to perform measurements) and the desire to use them directly in the natural environment, it is necessary to obtain sensors characterized by sufficiently good values of analytical parameters and, at the same time, easy to use and mechanically resistant [9]. Reliable measurement results can only be achieved with electrodes exhibiting a specific set of characteristics.

**Figure 1:**
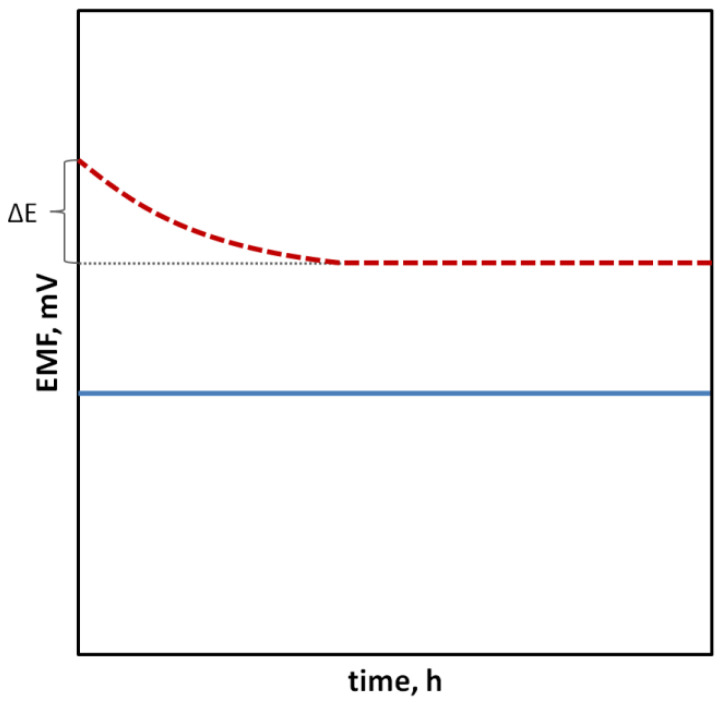
Change in the electrode potential immersed in the solution of the main ion in time, indicating electrode with more stable (⸺) and less stable (- - -) potential.

**Figure 2:**
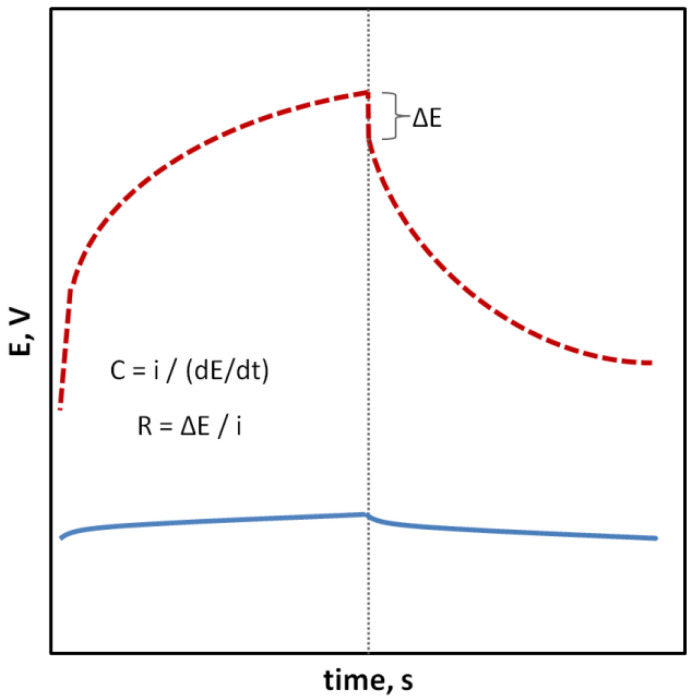
Chronopotentiogram representing an electrode with a stable (⸺) and less stable (- - -) potential; formulas for determining the electric capacitance (C) and total resistance (R), where potential drift (dE/dt), ΔE—potential jump as a result of changing the direction of the electric current (i).

**Figure 3:**
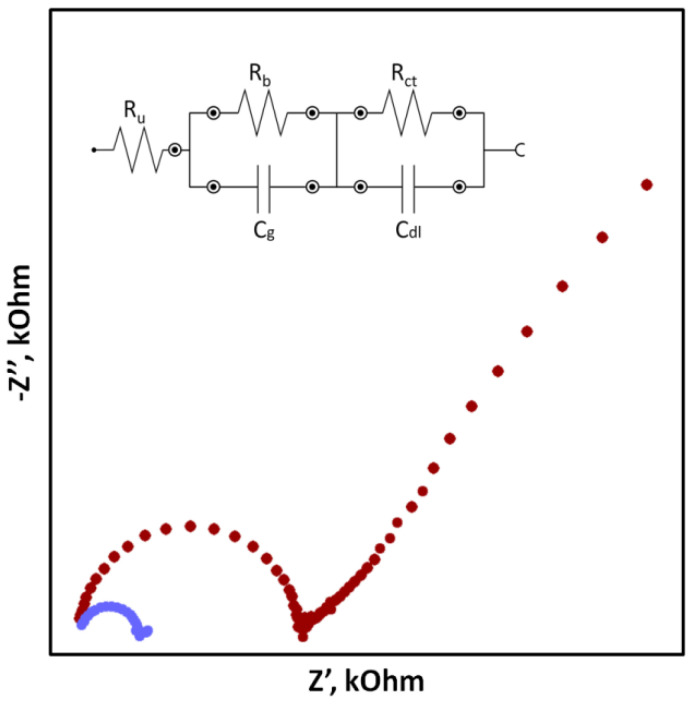
Exaplary impedance spectra for coated disc electrode without solid contact (red dots) and solid contact electrode (blue dots) with an electrical circuit (where R_u_uncompensated series resistance; R_b_-bulk resistance; C_g_-geometric capacitance; R_ct_-charge transfer resistance; C_dl_low-frequency layer capacitance).

In the case of SCISEs, potential stability, both short-term and long-term, is particularly important. Long-term stability can be determined by systematically measuring the potential of the sensors at constant intervals over an extended period of time (e.g., days, weeks and months). This parameter is usually characterized as the change in potential E^0^ over time. With regard to short-term stability, two approaches are possible. The first one consists of continuous measuring of the electrode potential in a relatively short time (1–3 h, that is the time needed to perform calibration and a series of measurements) in no-current conditions and determining the potential drift as ΔEMF/Δt (Figure 1). The second faster way uses the chronopotentiometry method (CP) and the measurement of the changing electrode potential in time (for example, 60 s) after applying a short-term electrical impulse (the most often in nA) to the electrode. The potential drift, which is a measure of stability, is determined from a rectilinear section of the chronopotentiometric curve from the dE/dt relationship. Based on the determined potential drift, the capacitance of the electrodes can be determined according to the equation C = i/(dE/dt). The chronopotentiometry technique can also be used to determine the resistance of the electrode, which often changes as a result of adding a solid contact material (Figure 2) [33]. Another technique commonly used to assess the effectiveness of a solid contact material is electrochemical impedance spectroscopy (EIS), an advanced method for studying electrode processes. In the case of ISEs, this method allows the determination of the membrane resistance, electric capacitance and charge transfer resistance between the ion-sensitive membrane and the internal electronic conductor [34,35,36]. These data are determined on the basis of the analysis of the impedance spectrum determined in a wide frequency range of 0.1 Hz–100 kHz by the semicircle method or by fitting an equivalent electrical circuit (Figure 3).

**Figure 4:**
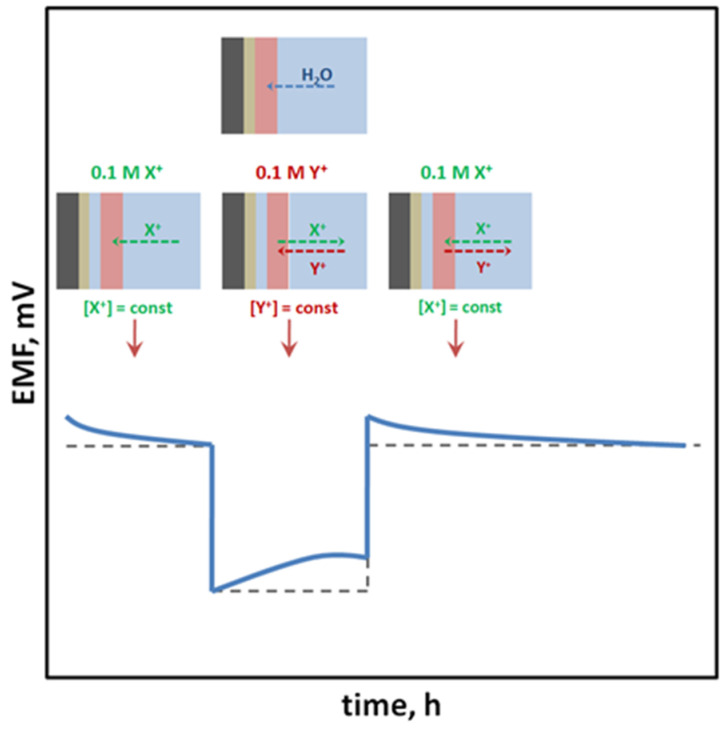
Results of the water layer test obtained for (⸺) electrodes with a tendency to form an undesirable water layer and (- - -) electrodes without this tendency.

SCISEs are tested for the formation of a water layer, which is formed as a result of water uptake and transport through the ion-selective membrane material [1]. The composition of the water layer may change during the modification of the sample solution composition in which the electrode is immersed, due to the penetration of interfering ions inside the layer, which is the reason for the drift of the measured potential [37]. This is a particularly undesirable process; therefore, water layer tests are performed (usually in accordance with the procedure proposed by Fibbioli et al. [37]) consisting in the use of high-concentration solutions of the main ion and interfering ion, and the observation of the potential drift caused by the change of solutions (in the order: main ion → interfering ion → main ion). Electrodes without a tendency to form a water layer maintain a constant potential after reaching a certain value after immersing the sensor in the interfering ion solution and quickly return to the initial potential value previously obtained in the solution of the main ion to which the membrane is sensitive (Figure 4).

## 3. Statistics

**Figure 5:**
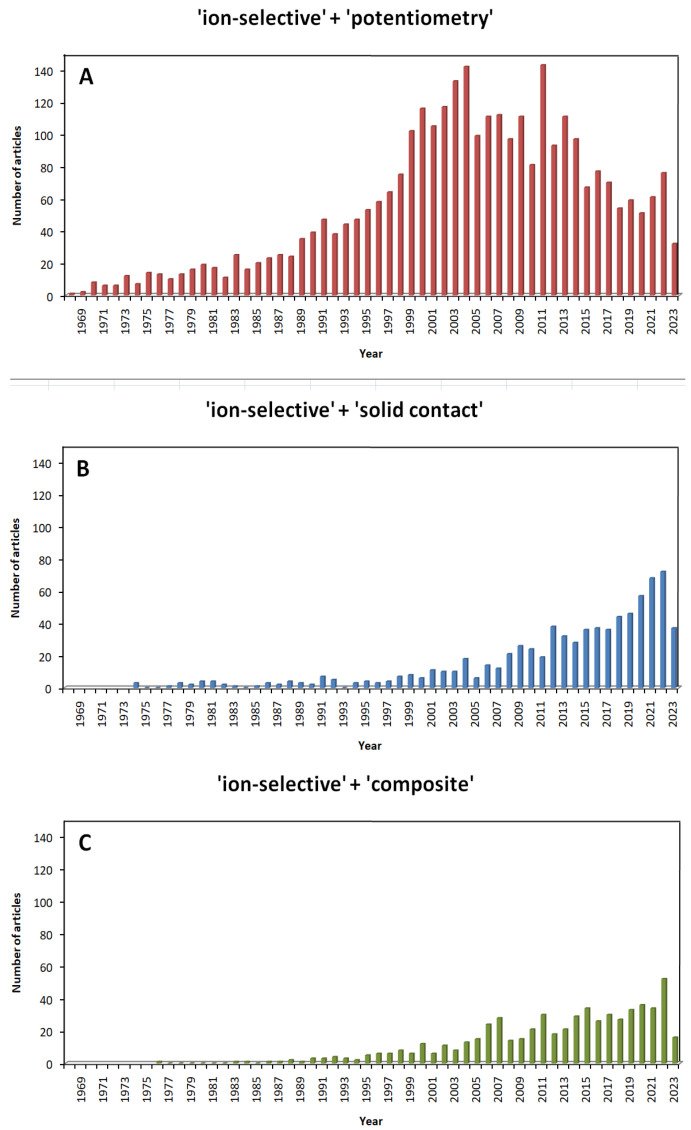
Results based on the SCOPUS database for scientific articles available after entering selected keywords (contained in “article title, abstract and keywords”) depending on the year of publication (access date: 21 May 2023): (**A**) ■ “ion-selective” + “potentiometry”; (**B**) ■ “ion-selective” + “solid contact” and (**C**) ■ “ion-selective” + “composite”.

The high popularity of potentiometric methods can be confirmed by the results of searching for scientific articles in databases. In the case of the SCOPUS database with the “search within article title, abstract, keywords” option, after entering the entries “ion-selective” and “potentiometry”, 3105 results appeared (as of 21 May 2023). Since the turn of the 20th and 21st centuries, articles on this subject appear regularly, often in excess of 100 papers per year, and the vast majority of these papers focus on SCISEs. The first mentions of “solid contact” appeared in 1974, and since the 1990s, the number of articles on this subject has been steadily increasing. In recent years, the construction of SCISEs has been dominated by the use of composite and/or multi-component materials (Figure 5).

## 4. Solid Contact Materials

SCISEs are simple and easy to use and do not require complicated and expensive equipment. The production of electrodes is also, in most cases, not a complicated or very expensive process, but the cost depends on the type of selected internal electrodes and the market price of materials used in the construction or the synthesis of these materials. In order to improve the analytical parameters of sensors, many materials have been used till now as solid contact. In most cases, this affected mainly the electrical parameters of the electrodes, in particular, increasing the capacitance of the double layer and reducing the membrane resistance. An important change resulting from the additional modification of the electrode construction is a significant improvement in the stability of their potential compared to unmodified electrodes [38,39]. Sometimes, analytical parameters are also improved, the range of linearity of the calibration curve can be extended [40,41] or the detection limit can be lowered [40,42]. Selection of the appropriate conductive material is particularly important due to the significant impact of the type of intermediate layer also on the stability of the composition of the ion-selective membrane [43].

**Figure 6:**
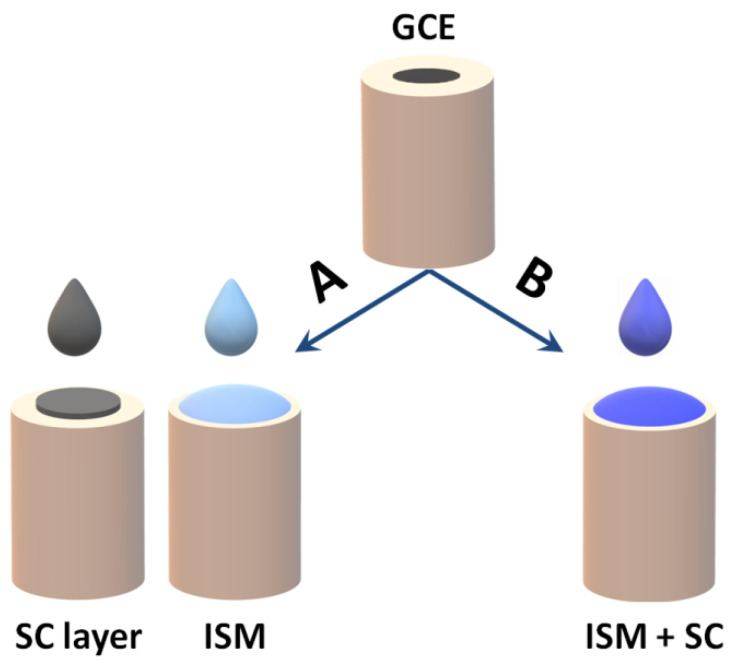
Scheme of SCISEs preparation: (A) 2 steps—the application of an SC layer and then covering it with an ISM layer and (B) 1 step—the direct application of ISM with SC additive.

Newly developed materials which could be used as solid contact should meet a number of requirements. Firstly, they must be materials with a high volumetric capacity ensuring potential stability and chemically stable, not generating any side reactions in the ion–electron transduction process. In addition, the conversion process between ionic and electronic conductivity should be reversible. It is also important that the material is sufficiently hydrophobic so that no water layer forms between the inner electrode material and the ion-selective membrane [44]. The choice of the material used as SC should be made taking into account many requirements and parameters, for example, based on the method and cost of their synthesis, their mechanical resistance, durability and analytical effectiveness and lifetime [31]. The way of applying the solid contact material is twofold. Most often, it is used as an intermediate layer between the electrode surface and the ion-sensitive membrane, which is applied to the substrate by drop casting or electrochemical deposition. The second way is to use a solid contact material as a component of the ion-selective membrane (Figure 6).

### 4.1. Conductive Polymers

The first materials used as solid contact were conductive polymers (CPs). From this group of compounds, polypyrrole (PPy) [45,46,47] was used for this purpose already in the 1990s, then also poly(3,4-ethylenedioxythiophene) (PEDOT) [48,49,50], poli(3-octylothiophen) (POT) [51,52,53,54,55], poly(N-methylpyrrole) (PNMP) [56] or polyaniline (PANI) [57,58,59]. Various types of conductive nanostructured polymers mainly based on polyaniline were also used for the construction of the sensors: in the form of nanotubes [60], nanofibers [61], nanoparticles [62] or nanowires [63,64].

The wide application of CPs is due to their numerous advantages: simplicity of synthesis and variety of possible modifications using other materials, high environmental stability, attractive price and unique chemical structures [65,66]. These compounds are characterized by double conductivity (ionic and electronic) and therefore can be successfully used as ion–electron transducers placed in the form of an intermediate layer of solid contact in ion-selective electrodes or as an additional component dissolved in the membrane or in a functionalized form as ready-made membranes [67].

The first sensors using CPs had significantly improved membrane conductivity but often were sensitive to changing environmental conditions (light, the presence of oxygen and carbon dioxide in the sample solution and changes in pH) as a result of side electrochemical reactions [66]. These materials also often did not show sufficiently high hydrophobicity, which resulted in the formation of a water layer between the membrane and the inner electrode. Therefore, the search for materials with similar properties, but differing in the method of synthesis, structure or composition and more hydrophobic, was started in order to improve the adhesion of the ion-selective membrane and reduce water absorption [9].

### 4.2. Carbon Nanomaterials

Carbon nanomaterials (CNs) are another group of materials with unique properties that have so far been commonly used in the construction of ion-selective electrodes. They are defined as materials with at least one dimension less than 100 nm [68]. They are particularly popular mainly due to their unique chemical, physical and electrical properties, i.e., mechanical resistance, hydrophobicity, good electrical conductivity and high electrical capacity [44,69]. The very high surface-to-volume ratio resulting from the small size of the nanostructures favors their interaction with neighboring materials, and their unique electrical properties improve potential stability, which makes them particularly attractive materials that can be used as ion–electron transducers in SCISEs [29]. The properties of the synthesized nanomaterials and their subsequent possible applications depend to a large extent on the method used for their synthesis [70,71]. In addition, these nanomaterials can be subjected to various types of modifications, e.g., by attaching selected functional groups to them in the process of derivatization in order to change their properties. In this way, it is possible to obtain nanomaterials characterized, for example, by better solubility or chemical reactivity compared to the starting materials [72,73,74]. Among the most popular carbon nanomaterials used in SCISEs are carbon nanotubes (CNTs) invented by Iijima in 1991. Numerous review articles refer to them [75,76,77,78,79,80,81,82,83,84], which describe methods of obtaining and purifying CNTs and their extensively studied properties, possible functionalization reactions and their use in the synthesis of nanocomposites. There are also many articles focusing on their use in electrochemistry [85,86,87,88].

Scientists have already described the results of research on the use of various types of carbon nanomaterials as a solid contact in the construction of electrodes: SWCNTs [69,89,90,91], MWCNTs [92,93,94,95,96], fullerenes [97,98], nanohorns [99], graphene [100,101] or colloid-imprinted mesoporous carbon (CIM) [39], amorphous carbon in the form of carbon black [102], three-dimensionally ordered microporous carbon (3DOM) [42,103] or chemically and electrochemically reduced graphene oxide (CRGO [104] and ERGO [105]).

### 4.3. Metal and Metal Oxide Nanoparticles

Currently, there are more and more studies on various types of nanoparticles (by definition, materials with at least two dimensions below 100 nm [68]) (mainly metal and metal oxide nanoparticles), their properties and possible applications [106]. One such application is the use of metal nanoparticles to improve the analytical parameters and electrical potentiometric sensors, which is possible due to their high redox activity and very large surface area. So far, metal nanoparticles have already been used for this purpose: gold [107,108,109,110], silver [111] and platinum nanoparticles [112,113,114]; metal oxides: copper(II) oxide [115], cerium oxide [116], ruthenium oxide [117] and iridium oxide nanoparticles [118], as well as bimetallic [119] or metal–nonmetal nanoparticles [120]. In addition to typical studies on the influence of the presence of nanoparticles on the direct operation of sensors, the use of silver nanoparticles with known bactericidal and antimicrobial properties [121,122] to extend the life of electrodes used to study complex environmental samples exposed to the presence of microorganisms and biofilms has also been described [123].

Metal oxides (PtO_2_, IrO_2_, RuO_2_, OsO_2_, Ta_2_O_5_ and TiO_2_) have been used in the construction of pH electrodes almost 40 years ago [124]. It has been confirmed that the modification of sensors with nanoparticles has a positive effect on the potential stability as a result of low resistance values and high capacitances achieved [116,117].

### 4.4. Nanocomposites

By definition, composites are materials that were created from at least two components different from each other, in order to significantly improve selected parameters characterizing the material and/or obtain new materials properties. The effective use of many single-component materials was the starting point for research on the development and use of composite materials in the field of ion-selective electrodes. Composites of carbon nanomaterials and polymers are particularly popular [24]. Addition, among others carbon nanotubes to polymers, makes it possible to obtain composite materials with improved mechanical, thermal, electrical, optical and chemical properties [24,25,125,126]. Composites obtained from polymers and carbon nanomaterials in combination with other materials, such as metal nanoparticles, metal oxides, ionic liquids and many others, are widely described [127,128,129,130]. Using various methods, scientists produce more and more composite materials that are a mixture of various types of components, for which, after research using a number of analytical methods (including Scanning and Transmission Electron Microscopy, Photoluminescence, UV-Vis and IR Spectroscopy, Raman Scattering, Diffuse Reflectance Spectroscopy, Energy-Dispersive X-Ray Spectroscopy, X-Ray Photoelectron Spectroscopy or X-Ray Diffraction), new applications are sought. These applications also include the use of newly synthesized materials as a solid contact material in SCISEs.

It is worth mentioning that nanocomposites based on conductive polymers with the addition of other materials or subjected to appropriate reactions are also used as an active substance added to the membrane mixture acting as a cation exchanger (often still in the case of electrodes with liquid contact, not described in detail in this review), among others PMMA-CeMoO_4_ nanocomposite [131], PANI-ZrI nanocomposite [132], [N,N′-bis(1-hydroxynaphthalene-2-carbaldehyde)-o-phenylenediamine] [133] and N,N’-bis(salicylaldehyde)phenylenediamine (salophen) [134].

Many examples of the use of composite materials as solid contact in ISEs have been described in the literature so far. An overview of such applications described in the literature in recent years is presented in the following subsections.

#### 4.4.1. Composites Based on Polymers and Carbon Nanomaterials

Composites made of various types of carbon nanomaterials and polymers have recently become very popular due to the advantages resulting from the combination of materials with significantly different properties. There are many methods to obtain this type of two-component nanocomposite, which can show much better mechanical resistance and increase electrical conductivity even by several orders of magnitude compared to its components separately [25]. This group of composites is dominated by materials obtained on the basis of conductive polymers and carbon nanotubes.

Nanocomposite obtained during the electrochemical synthesis of the conducting polymer poly(3,4-ethylenedioxythiophene) (PEDOT), where MWCNTs were used as dopants, was described in [135]. The electroactive film, PEDOT(CNT) was used as solid contact in K^+^-SCISEs. The electrical parameters of the electrodes were examined and sensors were also tested for sensitivity to changing measurement conditions. It was observed that the PEDOT(CNT) composite layer itself was sensitive to the presence of CO_2_, but after covering it with a polymer membrane, this sensitivity was no longer observed. The electrodes with the nanocomposite layer showed a low potential drift (12.0 µV s^−1^, i ± 1 nA), which was lower than the drift determined for the electrode with the Cl^–^ ion-doped PEDOT layer (17.6 µV s^−1^, i ± 1 nA). The effect of differences in low-frequency capacitance values was also noticeable (83 µF for ISE with PEDOT(CNT) and 57 µF for ISE with PEDOT(Cl)).

Electrodes sensitive to Ag^+^ ions using modified polypyrrole–multiwalled carbon nanotubes composite as SC with newly synthesized lariat ether as ionophore were described in publication [136]. Very good analytical parameters were obtained: a wide range of linearity 1.0 × 10^−7^–1.0 × 10^−1^ M, low detection limit—9.3 × 10^−8^ M and good selectivity of the sensors. In addition, they operated in a wide range of pH (1.6–7.7) for a minimum period of 50 days. Their practical application was tested on samples of silver sulphadiazine as a burning cream.

In article [137], Athavale et al. proposed sensors for the determination of ammonium ions in the in situ environment (eutrophic lakes). For the construction of the electrodes, composite polyvinyl chloride membrane impregnated with carbon nanotubes (CNT-PVC) and plasticizer-free methyl methacrylate–decyl methacrylate copolymer (MMA-DMA) was used. Profiles of the content of NH_4_^+^ ions were made depending on the depth of the water reservoir. The measurement platform, apart from potentiometric sensors, also had other sensors whose purpose was to measure additional parameters useful from the point of view of environmental research: conductivity, temperature, oxygen and photosynthetically active radiation.

A superhydrophobic polymer–carbon nanocomposite composed of a combination of carbon black with a highly porous acrylic-fluorinated graphene copolymer was proposed [138]. The material was characterized by a high capacitance of 1410 mF and a highly developed surface area, and the sensors constructed using it showed a Nernst slope of 59.1 mVdec^−1^ in the linearity range of 3.16 × 10^−7^–1 × 10^−1^ M and a low detection limit of 2.0 × 10^−7^ M. The use of numerous research methods (CP, EIS, cyclic voltammetry (CV), wettability measurements and tests of potential sensitivity to changing environmental conditions) confirmed that the use of the nanocomposite significantly improved the analytical parameters of the electrodes (potassium in this case).

A composite consisting of exfoliated graphene and polyaniline was used in Ca^2+^-SCISEs (PANI-graphene). The material was characterized by much better hydrophobicity than PANI (30° higher contact angle with water). The measured redox capacitance of the electrodes with the composite layer was 11 µF and with the PANI layer was 8 µF. Sensors with good stability and potential reproducibility, low detection limit (5.0 × 10^−8^ M) with no tendency to form a water layer between the substrate material and the ISM were obtained [101].

In publication [139], carbon paste electrodes with graphite-oxide-imprinted polymer composite for the determination of Cu^2+^ ions were described. The authors made a number of electrodes differing in the qualitative and quantitative composition of the mixture and selected the one for which the best analytical parameters were obtained (20.0% ionophore (Cu^2+^-ion-imprinted polymer), 10.0% paraffin oil, 5.0% MWCNTs and 65.0% graphite oxide). These sensors had a slope of calibration curve equal to 26.1 dec^−1^ in the linearity range of 1.0 × 10^−6^–1.0 × 10^−1^ M and an LoD of 4.0 × 10^−7^ M. The sensors worked fast (response time ~ 3 s) in the pH range of 4.0–8.0. While the range of linearity was not very wide, the authors ensured that the lifetime of the electrodes was more than 1 year, which is very promising in the case of potentiometric sensors. The obtained electrodes were used to determine the content of copper(II) ions in water samples (spiked river, dam and tap water) and were also used in potentiometric titration.

Nanocomposite of POT-MWCNTs (poly(3-octylthiophene-2,5-diyl) and multiwalled carbon nanotubes) with high capacitance, electrical conductivity and lipophilicity (in which POT acted as a dispersant) was used as SC in potassium all-solid-state electrodes [140]. Using numerous methods (contact angle and fluorometric measurements, Raman spectroscopy, etc.) the authors examined the properties of the nanocomposite and confirmed that the nanocomposite is characterized by a large contact angle and high conductivity, and its use prevents the unwanted process of POT transfer to the membrane phase, which eliminates the risk of changes in analytical parameters due to uncontrolled changes in the composition of the membrane. The slope of the obtained sensors was 56.3 mVdec^−1^ in the range of linearity 1 × 10^−6^–1 × 10^−1^ M (LoD ~ 1.6 × 10^−7^ M).

A nanocomposite membrane with the addition of MWCNTs was used in ISEs sensitive to Pb^2+^ ions based on gold disc electrodes [141]. The preparation of the electrodes was a simple one-step process, which consisted in dispersing MWCNTs in a plasticized ion-selective membrane in the ultrasonication process without the use of surfactants. Sensors with a slope of 29.0 mVdec^−1^, a linearity range of 2.0 × 10^−3^–2.0 × 10^−9^ M and a low detection limit of 4.0 × 10^−10^ M were obtained. In addition to typical potentiometric measurements, the contact angle test and chronopotentiometric measurements were also performed. The modification of the electrodes significantly reduced the potential drift of the electrodes (from 533.25 µV s^−1^ to 19.8 µV s^−1^, i ± 1 nA). According to the authors, the electrodes had very good long-term potential stability and no water layer and were also resistant to changing environmental conditions. The effectiveness of the sensors was checked on the basis of tests of tap water samples.

The planar sensors for the determination of NH_4_^+^ ions were described in [142]. The solid contact composite material in which graphite particles were embedded in a polyvinyl butyral matrix was used. The sensors operated in the pH range of 2.5–8.5 and reached a slope of 57.3 mVdec^−1^. Measurements for the electrodes were made in static and flow mode, and according to the authors, they can be successfully used for measurements in the in situ environment.

Calcium electrodes with a newly synthesized ionophore (4,7-diaza-2,3,8,9-dibenzo-15-crown-5) were described in [143]. A composite of carbon nanotubes and PVC was used as a solid contact. Studies were performed to optimize the composition of the ion-selective membrane, obtaining a low detection limit (9.1 × 10^−8^ M). The electrodes were used for potentiometric titration and testing of horticulture drain water samples using AAS as a comparative method.

In paper [144], ion-selective electrodes sensitive to chloride ions were described, in which a nanocomposite obtained from MWCNTs and PANINFs-Cl (polyaniline nanofibers doped with chloride ions) was used as the SC. Initial measurements of the electrical parameters (using CP and EIS) of the electrodes modified with an intermediate layer consisting of nanocomposites with different mass ratios of components and a layer containing only MWCNTs or PANINFs-Cl were made. It was confirmed that the nanocomposites showed better electrical parameters (lower resistance, higher capacitance) than their components separately. The best among the tested electrodes turned out to be sensors with an intermediate layer of PANINFs-Cl:MWCNTs nanocomposite in a weight ratio of 2:1. Comparing the SC layers, the layers containing (2:1) PANINFs-Cl:MWCNTs showed a capacitance almost 4 times higher than PANINFs and more than 10 times higher than MWCNTs. In the case of sensors, the electrodes with this layer of nanocomposites were characterized by almost 4 times lower membrane resistance and over 200 times lower charge transfer resistance with a simultaneous almost 200 times increase in the capacity of the double layer, as a result of much faster processes of diffusion and charge transport at the membrane/GCE. In addition, electrodes with a nanocomposite intermediate layer (2:1) PANINFs-Cl:MWCNTs showed a slope of characteristic equal to –61.3 mVdec^−1^, linearity range of the calibration curve 5 × 10^−6^–1 × 10^−1^ M and the lowest detection limit (2.3 × 10^−6^ M) and the best potential stability (0.03 mVh^−1^) compared to the other modified electrodes and the unmodified electrode. All sensors were resistant to changes in external conditions and maintained a constant potential in the pH range of 4–9.

GCE-based potentiometric sensors sensitive to cesium ions were described in publication [145]. The SC material was described as magnetic multiwalled carbon nanotubes/cesium-ion-imprinted polymer composite (MWCNTs@Cs-IIP). SEM and TEM images of both the nanocomposite and carbon nanotubes were presented. The process of optimizing the composition of ion-selective membranes with different types of plasticizers (DBP and NPOE) and different content of PVC and other components was performed. The electrode with the optimal composition showed a low detection limit of 4.0 × 10^−8^ M, but a relatively narrow measuring range of 1 × 10^−7^ to 1 × 10^−4^ M. The practical application of the sensors was tested on samples of river water, industrial wastewater and brine.

A multi-component combination of materials was proposed by Niemiec et al. [146] in the work about the modification of various carbon materials (electrospun carbon nanofibers (eCNFs), electrospun carbon nanofibers with embedded cobalt nanoparticles (eCNF-Co) and hierarchical nanocomposite with the nanoparticles of cobalt and nickel as a catalyst for the growth of carbon nanotubes (eCNF/CNT-NiCo)) using a POT polymer. Materials with an interesting structure were obtained, which, as a result of POT modification, were characterized by increased hydrophobicity. In the case of eCNFs and eCNF-Co, a significant increase in capacitance was obtained: from 1.62 to 7.87 mFcm^−2^ for eCNFs and from 2.67 to 4.37 mFcm^−2^ for eCNFs-Co, while for eCNF/CNT-NiCo effect was opposite and capacity was reduced. These materials placed as an intermediate layer allowed to obtain potassium sensors with low detection limits (3.2 × 10^−6^ M) and excellent potential stability (0.03 mVh^−1^ for the composite with eCNFs).

An interesting solution was presented by the authors in article [147], proposing ternary nanocomposites consisting of three types of materials: conductive polymer (poly(3-octylthiophene-2,5-diyl)), carbon nanomaterials (carbon black and nanotubes) and metal oxide (hydrated iridium dioxide). The obtained materials were characterized by high hydrophobicity (contact angle up to 180°). The obtained electrodes were characterized by very good long-term stability (potential drift: 43 µV h^−1^ for the NT-based electrodes and 79 μV h^−1^ for the CB-based electrodes) and high capacitance (1.5 and 0.9 mF, respectively).

In article [116], the same authors described the use of hydrous cerium dioxide nanoparticles and their combination with carbon nanotubes and poly(3-octylthiophene-2,5-diyl) as SC in potassium electrodes. The measured contact angles obtained for the tested materials confirmed that the combination of hydrophilic CeO_2_ nanoparticles (contact angle 17°) resulted in the formation of superhydrophobic composites (100° and 120° for hCeO_2_-NTs and hCeO_2_-POT) with a large surface area. The material with the highest capacitance turned out to be hCeO_2_-NTs (800 ± 10 µF), which also contributed to achieving the best potential stability by the sensors that were constructed using it. The proposed K^+^-SCISEs are able to work in variable external conditions and in a very wide range of pH (2.0–11.5).

#### 4.4.2. Composites Based on Polymers with Other Materials

The self-plasticizing triblock copolymer polystyrene-block-polybutadiene-block-polystyrene (PS-PB-PS, SBS), and a composite of this material and an ionic liquid (BMImPF_6_), was used in the construction of electrodes sensitive to a number of cations (Na^+^, K^+^, Ca^2+^, Cu^2+^ and Pb^2+^) on the gold electrode surface [148]. The authors confirmed that the membrane containing SBS is more hydrophobic than the PVC membrane, thanks to which the electrode does not show a tendency to form a water layer. Plasticizer-free electrodes with characteristic slopes similar to Nernst were obtained.

Poly(3,4-ethylenedioxythiophene) doped with poly(4-sodium styrene sulfonate), i.e., PEDOT(PSS), was used as SC in studies on the change in standard potential of electrodes (E^0^) [149]. Three types of ISM were applied on the layer of SC put previously on GCE: valinomycin-based potassium-selective membranes with and without ETH-500 (the lipophilic salt: tetradocedylammoniumtetrakis(4-clorophenyl)borate) and membrane without ionophore. Based on the results, it was found that the E^0^ of electrodes containing a conductive polymer as an ion–electron transducer can be moved by applying low-current pulses (nA) or by applying a potential that differs from the open-circuit potential of the sensor in the selected electrolyte solution. According to the authors, the ability to control E^0^ and its adjustment in a way that can be predicted or repeated can be used in the future in practical measurements.

SCISEs sensitive to ammonium ions were described in publication [58]. The obtained sensors were characterized by an immediate response time (<1 s) and a long lifetime (3 months). The electrodes were able to work in a very wide range of pH (2.6–10.1). Wastewater samples were tested using them and the obtained results were verified by comparing them with the results obtained by means of titration and the colorimetric method.

In publication [150], the authors proposed solution-casted chitosan/Prussian blue nanocomposite (ChPBN) as a solid contact in ISEs sensitive to sodium ions. Thorough morphological studies of the material confirmed that it has the structure of a macroporous chitosan network containing Prussian blue nanoparticles inside. The nanocomposite was placed as an intermediate layer between the ion-selective membrane and the screen-printed carbon electrode. The electrodes were characterized by the slope of the calibration curve of 52.4 mVdec^−1^ in the range of only 1 × 10^−4^–1 M, while the electric capacitance estimated by CP was 154 ± 4 μF and potential stability (potential drift of 1.3 μV h^−1^) was promising. Due to the high volumetric capacity of the synthesized material and its highly porous structure, a large interfacial surface was obtained in relation to the outer layer of the selective sodium membrane, which improved the stability of the electrode potential. The electrodes containing the nanocomposite layer showed better properties than both the unmodified electrodes (which is obvious) and the electrodes in which single components of the nanocomposite were used as SCs.

The next composites that were used as SCs were made by combining polypyrrole and zeolites with different SiO_2_:Al_2_O_3_ ratios, i.e., 23, 80 and 280 (PPy/H-ZSM composites) [151]. SEM pictures of the materials from above and in the cross-section position were taken, which showed the presence of zeolite both on the surface and inside the composite, and the anionic groups of the zeolite acted as counterions. Based on the contact angle tests, it was estimated that the hydrophobicity of the obtained composites decreased with the decrease in the Al_2_O_3_ content in the composite structure. Both the analytical and electrical parameters of the potassium electrodes made using them have been extensively studied. The slopes of the electrode calibration curves were in the range of 52.1–53.1 mVdec^−1^ and were comparable to the values obtained for electrodes based on PPy-Cl; however, for electrodes with a composite, lower detection limits were obtained.

The intermediate layers of SC deposited on GCEs composed of MOMs (molecular organic materials) such as tetrathiafulvalene-tetracyanoquinodimethane (TTF-TCNQ), tetrathiafulvalene (TTF) and its chloride (TTFCl) with/without carbon black were described by Pięk et al. [152]. SEM pictures of the materials and CV tests were taken. All modified chloride electrodes showed a slope in the range of −58.18 and −59.63 mVdec^−1^. The improvement in the electrical parameters of the electrodes was confirmed by CP and EIS measurements.

K^+^-SCISEs with SC of a nanocomposite composed of ruthenium dioxide and poly(3-octylthiophene-2,5-diyl) (RuO_2_+POT) have been described [153]. SEM images and contact angle measurements were taken for the nanocomposite layers (centrifuged ~149° and noncentrifuged ~110°), and much higher values were obtained than for its components separately (RuO_2_ ~18° and POT ~91°). The parameters of sensors with different SC layers were tested. The electrodes with the nanocomposite layer showed a resistance of 273.7 ± 0.5 kΩ and a capacitance of 1.167 ± 0.028 mF and were resistant to changes in external conditions and the formation of a water layer.

A highly hydrophobic material (with a water contact angle of 104°) prepared by electrospinning e-PANI-PS (hydrophobic polyaniline-polystyrene microfiber films) was used as a solid contact in Pb^2+^-SCISEs [154]. Electrodes with a very low detection limit (5 × 10^−9^ M) and a Nernst slope of the characteristic curve (29.1 mVdec^−1^) were obtained. The sensors showed a much lower resistance and a higher electric capacity compared to the sensors containing only PANI microfibers, and thanks to the high hydrophobicity of the SC material, they were not susceptible to the formation of a water layer. The content of Pb^2+^ ions in tap water samples was determined and compared with the results obtained using the graphite furnace atomic absorption spectrometry method (GFAAS). The results obtained at the concentration level of 10^−8^ M were similar to those obtained by the comparative method.

In article [155], the authors proposed miniaturized all-solid-state electrodes (5 mm diameter), created from the coating of Au electrodes with poly(3-octyl-thiophene) and molybdenum disulfide nanocomposite (POT-MoS_2_), which can be successfully used to monitor nitrate content in soil samples. Small-size reference electrodes coupled with miniaturized ISEs have also been constructed, which consist of a screen-printed Ag/AgCl electrode covered with a protonated Nafion layer (to prevent chloride ions (Cl^−^) leaching during measurements). The practical application of the construction was tested by immersing the sensor in soil suspension for almost a month, where the nitrate concentration was measured continuously.

In the construction of Pb^2+^-SCISEs, polyaniline doped with titanium dioxide (PANI-TiO_2_) was used as an intermediate layer of SC [156]. Using X-ray diffraction (XRD), the structure of the materials was investigated. A very low detection limit of 7.9 × 10^−10^ M was achieved. The obtained electrodes were resistant to environmental changes (presence of gases, change of lighting) and were with very good selectivity, which is particularly important in the possible testing of environmental samples. As part of the analytical application of SCISEs, three samples of tap water were tested, and the obtained results were compared with those obtained using an atomic absorption spectrometry (AAS) method.

In addition, the combination of poly(3,4-ethylenedioxythiophene) backbone with hydroquinone pendant groups (PEDOT-HQ) was used as solid contact in K^+^-SCISEs [157]. A number of tests of the stability of the sensors in variable external conditions (gases, light) were carried out. A low detection limit of 2.0 × 10^−7^ M and a close to the theoretical slope of the calibration curve of 60.9 mVdec^–1^ were obtained.

A new nanocomposite material—ruthenium dioxide and poly(3,4-ethylenedio xythiophene) polystyrenesulfonate composite—was used as SC in K^+^-SCISEs [158]. The nanocomposite was characterized by a very high capacitance (~17.5 mF), as was the electrode with a layer of this material (~7.2 mF). SC layers of different thicknesses were examined by CP, and then electrodes with optimal SC thickness were made. Sensors with good potential stability, Nernst slope, resistance to changes in external conditions and the formation of an undesirable water layer were obtained.

Lead electrodes with SC made of silver nanoparticles and polyaniline were described in article [159]. Several methods were used to test the sensors, including XPS, CV and EIS. A very low detection limit (6.3 × 10^−10^ M) and fast sensor response time (<5 s) were obtained. Tap water samples were analyzed using atomic absorption spectrometry (GFAAS) as a comparative method.

SCISEs for the determination of anions: Cl^−^ and NO_3_^−^ with a short response time were constructed using an electrodeposited composite layer of manganese dioxide and poly(allylamine) [160]. A number of studies were performed including X-ray diffraction (XRD), voltammetry, EIS and CP. Modified sensors were characterized by much better parameters than sensors with ISM applied directly to the Pt electrode material.

Application of the synthesized material EDOT-S (3,4-ethylenedioxythiophene and 4-(2,3-dihydrothieno[3,4-b][1,4]dioxin-2-yl-methoxy)-1-butanesulfonic acid, sodium salt) in the construction of SCISEs sensitive to potassium and calcium ions on a substrate with gold disk electrodes was described in [161]. Sensors with a slope of the calibration curve close to Nernst’s (57.2 and 28.5 mVdec^–1^ for K^+^ and Ca^2+^, respectively) were obtained. The obtained electrodes were used to determine the content of appropriate ions in the water sample from the stream, and the obtained results were compared with the values obtained by other analytical methods: ICP-OES (for K^+^) and colorimetry (for Ca^2+^). Promising results were obtained: 28.1 ± 1.05 and 28.0 ± 0.71 µM K+ and 257 ± 7.27 and 262 ± 1.5 µM Ca^2+^ (where the first result was obtained potentiometrically, and the second by comparative method).

The biocompatible and stable conductive material used, as the authors suggested, in bioelectronics was poly(3,4-ethylenedioxythiophene)-polyethylene glycol (PEDOT:PEG), which was used as SC in NO_3_^−^-SCISEs based on carbon (C110) screen-printed electrodes (SPEs) [162]. A simple fabrication method was employed, which involved applying a PEDOT:PEG mixture onto the surface of the working electrode, drying it, immersing the electrode in a solution of the selected salt (in order to carry out the ion-exchange process of the originally present ClO_4_^–^ ions) and subsequently applying the ISM. After drying and conditioning, sensors were obtained with good selectivity and a slope of the calibration curve maintained for a period of 72 days (change from −55.8 to −53.3 mVdec^−1^). In order to check the practical applicability of the electrodes, the content of nitrate ions was determined in agricultural medium samples, which had not been subjected to any pre-treatment. Satisfactory results were obtained (less than ±2% logarithmic deviation compared to the comparative method).

#### 4.4.3. Composites Based on Carbon Nanomaterials with Other Materials

A composite material in the form of carbon black supporting platinum nanoparticles (PtNPs-CB) was used as an SC in nitrate electrodes [163]. SEM and TEM images were taken and the size and distribution of Pt nanoparticles were estimated. Tests for the formation of a water layer of the sensors were also carried out, and the dependence of their potential on the redox potential of the sample solutions was examined. Low detection limits were obtained (7.9 × 10^−7^ M for K^+^-SCISEs and 5.0 × 10^−7^ M for NO_3_^−^-SCISEs). The authors confirmed that the long-term stability of the sensors with PtNPs-CB was much better than for electrodes in which the components of the composite were used as a solid contact separately.

The combination of nanomaterials (SWCNTs and graphene) with an ionic liquid (tetradodecylammoniumtetrakis (4-chlorophenyl) borate, ETH 500) was used as SC in electrodes sensitive to Cu^2+^ and Pb^2+^ ions [164]. Electrodes with very low detection limits (4.0 × 10^−9^ and 1.8 × 10^−9^ M, respectively) and with good selectivity were obtained. Impedance measurements and water layer tests were also performed.

SCISEs for the continuous measurements of the H_2_PO_4_^−^ ions concentration were described in publication [165]. The obtained polymer–MWCNTs nanocomposite was characterized by high hydrophobicity and, when added to the polymer membrane, increased its hydrophobicity. The contact angle of the membrane with the composite increased by 10° compared to the membrane without the composite and was 92.8 ± 0.3°. The electrodes made using the nanocomposite showed the Nernstian slope of the calibration curve and were not prone to the formation of a water layer. The sensors were used to study samples of eutrophicated water, using spectrophotometry as a comparative method. Very similar results were obtained: for SCISEs (504 ± 0.05 µM) and for UV-VIS (502 ± 0.08 µM) H_2_PO_4_^−^ ions.

The SCISEs with graphene–tetrathiafulvalene nanocomposite (graphene-TTF/TTF+) were described in article [166]. This material showed high capacity, both double layer and redox capacitance (which was confirmed by CP) and high hydrophobicity. For a better study of the effect of the addition of the nanocomposite, electrodes with graphene as SC were also constructed. The electrodes containing the nanocomposite were characterized by better analytical parameters compared to the electrodes with graphene: the slope −59.14 instead of –58.3 mVdec^–1^ and the linearity range 1 × 10^−6^–1 × 10^−1^ M instead of 3 × 10^−6^–1 × 10^−1^ M.

In article [167], a number of different types of solid contact layers were investigated, which included various combinations containing, among others, graphene and 7,7,8,8-tetracyanoquinodimethane (TCNQ) and its copper salt (TCNQ-Cu), including nanocomposites made of these ingredients. Both the analytical and electrical parameters of electrodes based on composite materials were better compared to electrodes based on TCNQ and TCNQ-Cu alone. For the electrode with the GR, TCNQ and TCNQ-Cu nanocomposite, the detection limit was 2.5 × 10^−9^ M and the linearity range was 1.0 × 10^−8^–1.0 × 10^−2^ M. In terms of electrical parameters, compared to the basic electrode without SC, the resistance of the proposed electrode decreased almost 8 times (from 1251 to 161 kΩ), capacitance increased almost 200 times (from 2.14 to 396 µF), while potential drift decreased over 180 times (from 4660 to 25.2 µV s^−1^). The obtained sensors were successfully used to determine the content of copper(II) ions in water samples and packaging of food products, determining recovery and using voltammetry as a comparative method.

As proposed by Li et al., the three-dimensional porous graphene-mesoporous platinum nanoparticles composite (3D PGR-MPN) material was characterized by high hydrophobicity, conductivity and capacity of the double layer (1.4 mF) [168]. The obtained composite was also examined using SEM (scanning electron microscopy), HRTEM (high-resolution transmission electron microscopy), EDS (energy dispersive spectroscopy) and XPS (X-ray powder diffraction). It was used to construct electrodes sensitive to Cd^2+^ ions, which were additionally investigated by CV and EIS. Electrodes with a very low detection limit (1.6 × 10^–9^ M) were obtained. The electrodes showed no tendency to form a water layer and were insensitive to changes in lighting and gas content in solutions.

In paper [113], a two-fold application of graphene supporting platinum nanoparticles was described. This composite was used as a solid contact in K^+^-SCISEs, which were characterized by a Nernst slope of the calibration curve (59.1 mVdec^–1^) and good stability and potential reversibility, and in voltammetric sensors for paracetamol determination in the concentration range from 0.02 to 2.2 μM.

Bimetallic nanoparticles (AuCu) coupled with multiwalled carbon nanotubes (MWCNTs) were also used as a solid contact in ISEs [119]. This time in two types of ion-sensitive electrodes (Ca^2+^ and SO_4_^2–^ ions) for which the slope of the calibration curves was 29.0 and 27.0 mVdec^−1^, respectively, and the linearity ranges were 1.0× 10^−6^–1.0 × 10^−1^ and 1.0 × 10^−5^–1.0 × 10^−1^ M. The electrodes were also characterized by fast charge transfer (resistance 2.22 and 0.46 MΩ) and high double-layer capacitance (54 and 105 µF). The sensors were then used in practical determinations of ions in water and milk samples.

The nanocomposite MWCNTs:THTDPCl (ionic liquid: trihexyltetradecylphosphonium chloride) was used for the construction of GC-based electrodes sensitive to nitrate(V) ions [169]. The interaction of the cations of the ionic liquid with the surface of the MWCNTs ensures electrostatic and steric stabilization, which enables the formation of a homogeneous nanocomposite material that is easily dispersed in the polymer membrane material. In research nanocomposites obtained from various types of carbon nanotubes of different sizes (length and diameter) were used. As a comparative system, unmodified electrodes (ion-selective membrane without the addition of nanocomposite) and electrodes with solid contact in the form of an intermediate layer of MWCNTs were tested. It was found that the type of nanotubes in the nanocomposite had a significant impact on the parameters of the obtained electrodes, and the best results were obtained for the nanocomposite with MWCNTs, which were the shortest and most homogeneous among all others. The result of this was the synthesis of the most homogeneous membrane. Compared to the unmodified sensors, the electrodes containing the nanocomposite in the membrane were characterized by an order of magnitude greater linearity (1 × 10^−6^–1 × 10^−1^ M), a lower limit of detection (5 × 10^−7^ M) and also a higher slope of the calibration curve (−57.1 mVdec^−1^). The conducted water layer test showed that when the solutions were changed, the potential drift of the modified electrodes was much smaller than that of the unmodified electrode. It was also shown that the use of nanotubes in the form of a nanocomposite as a component of ISM is more effective than using them as an intermediate layer between the membrane and GCE. An additional advantage of the use of the nanocomposite was a simpler one-step method of electrode preparation.

The next publication on copper electrodes focused on the influence of the type of solid contact on the sensors’ behavior. The main object of interest in these studies was the nanocomposite MWCNTs:BMImPF_6_ (ionic liquid: 1-butyl-3-methylimidazolium hexafluorophosphate) in a weight ratio of 1:5 [170]. The interaction of the components of the composite results in its steric and electrostatic stabilization. This prevents aggregation of composite nanostructures and allows for uniform dispersion in the polymer phase of the ion-selective membrane. The sensors with the nanocomposite content in the membrane equal to 0, 2, 4, 6 and 8 wt.% were prepared. The modified electrodes were characterized by a higher slope and lower detection limits compared to the unmodified electrode and the electrode with liquid contact. The addition of 6% of the nanocomposite turned out to be the best in this case. The response time of the modified electrode was shorter, and the reversibility and stability of the potential were significantly improved. The potential drift decreased from 0.16 to 0.046 mVmin^–1^. The water layer test and the tests of potential stability depending on the change in the redox potential or the presence of gases were performed. After three months of use, the slope of the calibration curve slightly decreased (from 29.8 to 28.6 mVdec^–1^). The modification of the membrane with the addition of a nanocomposite resulted in improved analytical parameters. The determined membrane resistance of the sensors decreased more than 10 times (from 309 to 0.36 kΩ), and the capacitance in the low-frequency range increased more than 35 times (from 1.29 to 45.7 µF).

Nanocomposite materials consisting of ruthenium oxide and carbon nanomaterials (single-layer graphene, carbon black and multiwalled carbon nanotubes) with high capacitance (up to about 14 mF for MWCNTs + RuO_2_) and very low hydrophilicity were used in the construction of potassium electrodes [171]. The properties of the obtained nanocomposites as well as unmodified carbon nanomaterials were examined, confirming that the addition of RuO_2_ improves the electrical parameters of the materials. Sensors with very good stability and potential reversibility were obtained, with linearity in the range of 10^−6^–10^−1^ M, resistant to the formation of an undesirable water layer.

Studies on the construction of Fe^2+^-SCISEs using MWCNTs–gemifloxacin composite were described in publication [172]. The resulting sensor had a wide range of linearity (1 × 10^−2^–1 × 10^−8^ M) with a slope of 30.37 ± 0.3 mVdec^−1^ and a fast response time (5 s). Practical determination was carried out in samples of water, milk and multivitamin tablets, obtaining comparable results.

Comparative studies of materials that can be used as SCs in SCISEs have been described also in [173]. The study involved, among others, electrospun carbon nanofibers with/without incorporated cobalt nanoparticles and hierarchical nanocomposites of carbon nanotubes deposited on nanofibers with different cobalt/nickel nanoparticles. Both SEM pictures of the materials were taken and contact angles were measured to determine their degree of hydrophobicity. Electrodes with good potential stability, no tendency to form a water layer, resistance to changes in lighting and operating in a wide range of pH (2.0–10.5) were obtained. The best parameters were for SCISEs containing a layer of carbon nanofibers with high-density NiCo nanoparticles: detection limit of 5 × 10^−7^ M, linear range of 1 × 10^–6^–1 ×10^−1^ M and the highest potential capacity and reproducibility among all electrodes. The highest hydrophobicity was found in the layer of carbon nanofibers with carbon nanotubes and cobalt nanoparticles (contact angle 168°), and the electrodes with this SC layer showed the best reversibility and potential stability. The sensors were used to determine the content of potassium ions in the tomato juice samples and the recovery percentage was determined.

A smartphone-compatible portable system capable of real-time determination of Ca^2+^ ions in biological fluids using polystyrene-graphite nanoplatelets and carbon black as a solid contact was described in [174]. A series of SEM images of the tested materials and optimization of the qualitative and quantitative composition of the ion-selective membrane were taken. The electrical parameters of the electrodes (capacity 47.5 µF) were also measured, and tests of sensitivity to gases (CO_2_ and O_2_) and changes in lighting were performed. According to the authors, the production cost of the platform is low (<$25), but it is possible to test samples with very small volumes (<10 µL) in a short time (response time < 5 s). Tests in urine, artificial serum and artificial cerebrospinal fluid were successfully performed.

The highly stretchable 3D graphene oxide–carbon nanotubes composite was used in the construction of electrodes sensitive to ammonium ions with a slope of 59.6 mVdec^−1^, good potential reversibility and no tendency to form a water layer [175]. The nanocomposite layer was deposited on the electrode as a result of the electrodeposition process. The main application of the sensors is the determination of NH_4_^+^ ions in sweat to monitor the health of patients.

An interesting solution was presented in article [176], which describes fully flexible and wireless detection systems for Na^+^ ions, which integrates gold–carbon nanotube–gold sensors (Au/CNT/Au), with the use of which the calibration curve slope of 55.5 ± 0.3 mVdec^−1^ was obtained. This device is a good response to the miniaturization trend that has been popular in recent years. The production is a combination of the drop-casting method and electrochemical deposition method, and in addition to the ISE, an integrated miniaturized reference electrode was also prepared. The thin-film nanocomposite sensor showed high stability and mechanical strength.

Application as solid contact composite aerogel fabricated of cellulose fibers and carbon nanotubes in a homogeneous dispersion process was described in article [177]. The authors investigated the properties of the resulting composites using a number of analytical methods, including FTIR, SEM and thermal analysis. Sensors sensitive to potassium ions showed a good slope of the calibration curve equal to 52.0 mVdec^–1^, unfortunately in the range of only 1 × 10^−4^–1 × 10^−1^ M.

Pb^2+^-SCISEs electrodes obtained by adding a nanocomposite of carbon nanofibers (CNFs with a 100 nm diameter and a 20–200 µm length) and 1-hexyl-3-methylimidazolium hexafluorophosphate (ionic liquid: HMInPF_6_) to a polymer membrane were presented in article [178]. Studies were carried out to optimize the content of the nanocomposite in the ion-selective membrane (0–9% *w*/*w*) in two types of substrate electrodes: a platinum wire and a glassy carbon disc electrode. The analytical and electrical parameters of the electrodes containing the nanocomposite in the membrane were significantly improved compared to the unmodified electrodes. The best results were obtained for the Pt-based electrode with a membrane containing 6% of the nanocomposite addition. The determined value of bulk resistance decreased from 762 to 46.5 kΩ and charge transfer resistance from 9832 to 28.6 kΩ as a result of the addition of the nanocomposite. Electrodes with excellent selectivity (significantly better than electrodes without nanocomposite) and low detection limit (6 × 10^−9^ M) were obtained, with the use of which it was possible to successfully determine the content of lead ions in the certified reference material of wastewater (CRM SPS-WW1 Batch 113) with a result of 98.3 ± 3.8 µgL^−1^ for a given value of 100 ± 0.5 µg L^−1^.

In work [179], a nanocomposite of copper(II) oxide nanoparticles and multiwalled carbon nanotubes was used as solid contact of electrodes sensitive to Cu^2+^ ions. The sensors were also constructed using the components of the nanocomposite separately. The solid contact material was placed both as an intermediate layer and as a component of the membrane mixture. The electrodes with the nanocomposite in the membrane showed the lowest detection limit among all the tested electrodes (1.5 × 10^−8^ M) and the best short-term (132.0 µV h^−1^) and long-term potential stability (slope change during 8 weeks, 30.05 ± 0.09 mVdec^−1^). Based on the EIS measurements, it was found that the low-frequency capacitance of electrodes with SC material in the membrane was significantly higher for the addition of nanocomposite (92.5 µF) than for nanoparticles and carbon nanotubes (8.4 and 45.9 µF, respectively). The sensors were used to determine the content of copper ions in tap and mineral water samples.

#### 4.4.4. Other Composites

Ammonium electrodes constructed using SiO_2_/ZrO_2_/phosphate-NH_4_^+^ composite were described in [180]. The preparation of the composite was quite time-consuming, but low detection limits (1.6 × 10^−7^ M) were obtained. The electrodes were used to measure NH_4_^+^ ions in natural water samples.

Au and Pt nanostructures deposited by the electrochemical method (according to the authors, a process lasting only 6 min) played the role of SC in electrodes sensitive to Li^+^ ions [181]. The SC layer consisted of Au nanocorals and Pt nanoflowers together and separately. The finished materials were characterized by high hydrophobicity, high double-layer capacity and developed surface.

The material made from tetrathiafulvalene and tetracyanoquinodimethane (TTF-TCNQ) was described in article [182]. It was used as the SC in K^+^-SCISEs and NO_3_^–^-SCISEs, and slopes close to Nernst’s were obtained (58.52 and 58.47 mVdec^−1^, respectively). The solid contact material was examined using additional techniques, such as SEM or UV-VIS spectroscopy, and the electrode potential in solutions with different redox potentials was measured. The electrical parameters of the sensors were estimated using the CP method as well. High capacitance (255 and 629 µF) and low resistance (166 and 89 kΩ) were obtained for them. These parameters were significantly improved compared to SC-free ISEs.

The structures of potassium and nitrate electrodes were described in [183]. Conductive metal-organic frameworks (MOFs) were used as an SC, thanks to which sensors with high capacitance (204 µF) and good potential stability (drift of potential 11.1 µA h^–1^) were obtained. The electrodes did not show a tendency to form a water layer.

Electrodes with SC, resistant to changes in external factors and to the formation of a water layer, in which MoS_2_/Fe_3_O_4_ composite was used as SC, were also described in article [184]. For the construction of the electrodes, GCEs were used, on which a nanocomposite suspension obtained by the solvothermal method was dripped and then covered with a layer of membrane mixture. The resistance estimated for the electrode with the nanocomposite layer was 0.36 MΩ and was lower than for the electrodes with the MoS_2_ layer (0.47 MΩ) and also electrodes without the SC layer (0.52 mΩ). The potential drift rates were 2.9, 60.0 and 457.1 µV s^–1^ (for i = ±1 nA), and as expected, the electrodes with a MoS_2_/Fe_3_O_4_ layer exhibited the smallest drift.

In article [185], the authors proposed the use of the CoNiFe_2_O_4_ magnetic nanocomposite in Ce^3+^-SCISEs. X-ray diffraction and FTIR analysis methods were used to investigate the properties of the nanocomposite, such as purity and crystallization. A very wide range of linearity of the curve (1.0 × 10^−8^–1.0 × 10^−1^ M) and a low limit of detection (7 × 10^−9^ M) were obtained at the unfortunately quite low slope of the electrode characteristics (17.5 mVdec^−1^). However, the sensors can operate in a very wide pH range of 2–10. According to the authors, the electrodes were prepared from graphite rods of batteries, on which membrane mixtures with the addition of a nanocomposite suspension in THF were dripped. Sensors containing benzo-15-crown-5 as an active substance were characterized by very good selectivity. They were used to determine samples of water, milk and Coca-Cola, estimating recovery (at the level of 85–95%).

## 5. Comparative Studies

In Table 1, basic analytical parameters for electrodes prepared with composite materials are presented. One of the parameters significantly influenced by the appliance of solid contact is the stability of the potential. As mentioned in the introduction, the stability of the SCISEs’ potential can be assessed in two ways: potentiometric under the no-current condition and constant-current chronopotentiometry under polarizing conditions. The method of determining the potential drift is marked in brackets with the current value. As can be seen in Table 1, composite-based electrodes showed good potential stability (small value of potential drift). It is difficult to reliably compare the effectiveness of the described composites in improving the potential stability because the reported drift values were determined under different conditions (different measurement time, different ion concentrations in the solution for no-current conditions) and different current values in the case of constant-current chronopotentiometry. Among the data determined for i = 0, the lowest potential drift values of several dozen µVh^−1^ were obtained for electrodes based on ChPBN nanocomposite [150], graphene-TTF/TTF [166], PtNPs-CB [163], MOFs [183] and oAuCuNPs-MWCNTs [119]. Apart from the improved potential stability, the authors very often report the lowering of the detection limit and the extension of the measurement range as a result of the use of composite materials. Similar results have also been reported for electrodes based on single-component SC.

## 6. Conclusions

This has been a review of the use of composite materials in the construction of all solid-state ion-selective electrodes. Based on the summary presented in Figure 5, which shows the number of articles over time, it can be concluded that the interest in composite materials in this area has increased significantly in recent years. Based on the overview of more than 60 different composite materials, it can be observed that the overwhelming majority of them have been used as a solid contact in PVC-based polymeric membrane electrodes. Many new composite materials have been developed, based on materials used in the construction of electrodes separately, i.e., conductive polymers, carbon nanomaterials and others. Composites combine the desired properties of ingredients, which allows to obtain a material with better parameters (higher electric capacitance, lower resistance, larger surface area and more hydrophobic character). Thanks to this, the use of a composite is more effective than a single component. Many authors demonstrated in their works that composites show significantly better electrical parameters, i.e., higher electric capacitance and lower resistance. For some materials, very high capacitance values of the order of mF [158] are quoted. Interestingly, there is no simple proportionality between capacitance and SCISE potential stability. Thus, electrodes significantly different in capacitance show similar potential stability [144,158]. This proves that capacitance is an important parameter affecting the stability of the electrode potential, but other factors such as the composition of the membrane, the type of ion to which the electrode is sensitive and the substrate material also affect this parameter. Composites, such as single-component materials, are used in two ways, as an intermediate layer placed between the ion-selective membrane (ISM) and the electrode substrate (this method is most common) or as a component of the membrane. First of all, they perform the basic function as an ion to electron transducer, and additionally, other benefits are obtained. In most cases, the electrodes containing the composite material showed a lower limit of detection and a better slope of the characteristic. Nanocomposites based on carbon nanomaterials and ionic liquids improved the electrode selectivity [170,178]. Moreover, such composites show a much lower tendency to agglomeration than CNs and do not require the use of additional dispersants [169,170,178]. A similar effect was observed for MWCNTS-POT [140] and MWCNTs-CuONPs [179]. In some cases, the composite acts as an active potential-creating component, and it is simply added to an ion-selective membrane [145] or acts as an active component in paste electrodes [172,180]. Taking into account the type of ion to which obtained electrodes with the use of composites were sensitive, it can be seen that much more articles have been devoted to ISEs sensitive to cations. This is in line with the general trend for all ion-selective electrodes. In the group of cationic electrodes, K^+^-ISEs have been described the most, while NO_3_^–^-ISEs dominate among anion-sensitive electrodes. This is understandable, because when examining a new electrode material, it is justified to use a model membrane with a well-known response mechanism, as is the case with valinomycin-based ISM sensitive to K^+^ and TDMANO_3_-based ISM sensitive to NO_3_^−^. In summary, it can be said that the addition of composite materials was another significant step in the development of potentiometric sensors. This area will certainly continue to develop towards multi-component materials designed with specific properties in mind. This is facilitated by the continuous progress in materials engineering.

## Figures and Tables

**Table 1:** Summary of analytical parameters for electrodes described in the literature with a solid contact, ordered according to the type of ion.

Ion	Ionophore	Solid Contact	Slope, mVdec^–1^	LoD, M	Linearity Range, M	pH Range	Potential Stability	Ref.
NH_4_^+^	SiO_2_/ZrO_2_/phosphate-NH_4_^+^ composite	graphite powder	31.3	1.6 × 10^−7^	7.7 × 10^−7^–4.0 × 10^−2^	6.0–7.0	-	[180]
NH_4_^+^	ammonium ionophore I	CNT−PVC composite MMA−DMA copolymer	50.9 50.7	2.6 × 10^−7^ 2.2 × 10^−7^	1.0 × 10^−6^–1.0 × 10^−3^	-	<1000 µVh^−1^ (i = 0) 3600 µVh^−1^ (i = 0)	[137]
NH_4_^+^	ammonium ionophore I	CPANI	54.2	1.0 × 10^−6^	1.0 × 10^−4^–1.0 × 10^−1^	2.6–10.1	-	[58]
NH_4_^+^	ammonium ionophore I	graphite–PVB composite	57.3	4.8 × 10^−6^	1.0 × 10^−5^–1.0 × 10^−1^	2.5–8.5	-	[142]
NH_4_^+^	ammonium ionophore I	3D graphene–CNT	59.6	1.0 × 10^−6^	1.0 × 10^−6^–1.0 × 10^−1^	-	-	[175]
Li^+^	lithium ionophore VI	AuNanocorals/PtNanoflower	60.4	~1.0 × 10^−5^	1.3 × 10^−5^–1.0 × 10^−1^	-	5.2 µVs^−1^ (i ± 5 nA)	[181]
Na^+^	Na(X)	SBS-BMImPF_6_	58.2	-	1.0 × 10^−6^–1.0 × 10^−1^	-	-	[148]
Na^+^	sodium ionophore X	Au/CNT/Au	55.5	-	1.0 × 10^−3^–1	-	-	[176]
Na^+^	sodium ionophore VI	ChPBN nanocomposite	52.4	-	10^−4^–1	-	1.3 µVh^−1^ (i = 0), 288 µVs^−1^ (i = ± 100 nA)	[150]
K^+^	potassium ionophore I	PEDOT(CNT) composite	57.1	-	1.0 × 10^−6^–1.0 × 10^−1^	-	12.0 µVs^−1^ (i ± 1 nA)	[135]
K^+^	potassium ionophore I	SBS-BMImPF_6_	52.2	-	1.0 × 10^−6^–1.0 × 10^−1^	-	-	[148]
K^+^	potassium ionophore I	PEDOT(PSS)	60.1	-	1.0 × 10^−5^–1.0 × 10^−1^	-	690 µVh^−1^ (i = 0)	[149]
K^+^	potassium ionophore I	CB-FP	59.1	2.0 × 10^−7^	3.2 × 10^−7^–1.0 × 10^−1^	-	20.9 µVs^−1^ (i = ± 1 µA)	[138]
K^+^	potassium ionophore I	PPy/H-ZSM-5	54.2	7.1 × 10^−6^	1.0 × 10^−5^–1.0 × 10^−2^	-	130 µVh^−1^ (i = 0)	[151]
K^+^	potassium ionophore I	TTF-TCNQ	58.5	4.0 × 10^−7^	1.0 × 10^−6^–1.0 × 10^−1^	-	42.2 µVs^−1^ (i = ± 10 nA)	[182]
K^+^	potassium ionophore I potassium ionophore II	MOFs	58.2 54.1	5.0 × 10^−7^ 6.8 × 10^−6^	1.0 × 10^−6^–3.2 × 10^−3^ 3.2 × 10^−5^–3.2 × 10^−2^	-	15.0 µVs^−1^ (i ± 1 nA)	[183]
K^+^	potassium ionophore I	PtNPs-GR	59.1	3.2 × 10^−7^	1.0 × 10^−6^–1.0 × 10^−1^	-	-	[113]
K^+^	potassium ionophore I	MWCNTs:POT nanocomposite	56.3	1.6 × 10^−7^	10^−6^–10^−1^	-	-	[140]
K^+^	potassium ionophore I	RuO_2_ + POT nanocomposite	58.6	1.3 × 10^−7^	1.0 × 10^−6^–1.0 × 10^−1^	-	86 µVs^−1^ (i ± 100 nA)	[153]
K^+^	potassium ionophore I	PEDOT-HQ	60.9	2.0 × 10^−7^	1.0 × 10^−6^–1.0 × 10^−1^	-	100 µVh^−1^(i = 0)	[157]
K^+^	potassium ionophore I	GR + RuO_2_ CB + RuO_2_ NT + RuO_2_	58.95 58.03 58.25	-	10^−6^–10^−1^	-	120 µVs^−1^ (i = ± 100 nA) 240 µVs^−1^ (i = ± 100 nA) 200 µVs^−1^ (i = ± 100 nA)	[171]
K^+^	potassium ionophore I	MoS_2_/Fe_3_O_4_ nanocomposite	55.2	6.3 × 10^−6^	1.0 × 10^−5^–1.0 × 10^−1^	-	2.9 µVs^−1^ (i = ± 1 nA)	[184]
K^+^	potassium ionophore I	RuO_2_-PEDOT:PSS	58.9	-	1.0 × 10^−6^–1.0 × 10^−1^	3.5–10.0	14.3 µVs^−1^ (i ± 100 nA) 77 µVh^−1^ (i = 0)	[158]
K^+^	potassium ionophore I	hCeO_2_ hCeO_2_ + NTs hCeO_2_ + POT nanocomposites	55.3 58.9 58.2	-	1.0 × 10^−5^–1.0 × 10^−1^ 1.0 × 10^−6^–1.0 × 10^−1^ 1.0 × 10^−6^–1.0 × 10^−1^	2.0–11.5	86 µVh^−1^ (i = 0), 6000 µVs^−1^ (i = ± 10 nA) 95 µVh^−1^ (i = 0), 2300 µVs^−1^ (i = ± 100 nA) 240 µVh^−1^ (i = 0), 2700 µVs^−1^ (i = ± 100 nA)	[116]
K^+^	potassium ionophore I	eCNF, eCNF-Co eCNF/CNT-NiCo with POT nanocomposites	59.7 59.9 59.8	6.3 × 10^−7^ 1.3 × 10^−6^ 3.2 × 10^−6^	10^−6^–10^−1^ 10^−5^–10^−1^ 10^−5^–10^−1^	-	30 µVh^−1^ (i = 0), 30 µVh^−1^ (i = 0), 60 µVh^−1^ (i = 0)	[146]
K^+^	potassium ionophore I	EDOT-S	57.2	1.7 × 10^−6^	1.0 × 10^−5^–1.0 × 10^−1^	-	-	[161]
K^+^	potassium ionophore I	CNT/POT/hIrO_2_ CB/POT/hIrO_2_	57.3 58.8	-	1.0 × 10^−6^–1.0 × 10^−1^	3.5 –10.5	43 µVh^−1^ (i = 0) 79 µVh^−1^ (i = 0)	[147]
K^+^	potassium ionophore I	eCNF/CNT[HD]-NiCo	59.4	5.0 × 10^−7^	1.0 × 10^−6^–1.0 × 10^−1^	2.0–10.5	60 µVh^−1^ (i = 0) 31 µVs^−1^ (i ± 10 nA)	[173]
K^+^	potassium ionophore I	CA	52.0	8.5 × 10^−3^	1.0 × 10^−4^–1.0 × 10^−1^	-	-	[177]
Cs^+^	MMWCNTs@Cs-IIP composite		59.5	5.0 × 10^−8^	1.0 × 10^−7^–1.0 × 10^−4^	4.0–6.5	-	[145]
Ag^+^	lariat ether	MWCNTs–PVC composite	59.4	9.3 × 10^−8^	1.0 × 10^−7^–1.0 × 10^−1^	1.6–7.7	-	[136]
Ca^2+^	AU-1	SBS-BMImPF_6_	29.8	-	1.0 × 10^−6^–1.0 × 10^−1^	-	-	[148]
Ca^2+^	calcium ionophore I	PANI–graphene composite	28.7	5.0 × 10^−8^	3.0 × 10^−7^–1.0 × 10^−4^	-	5.2 µVs^−1^ (i ± 1 nA)	[101]
Ca^2+^	calcium ionophore IV	oAuCuNPs-MWCNTs	29.0	6.0 × 10^−7^	1.0 × 10^−6^–1.0 × 10^−1^	-	15 µVh^−1^ (i = 0)	[119]
Ca^2+^	calcium ionophore II	TPEs + carbon black	31.2	1.0 × 10^−5^	1.0 × 10^−4^–1.0 × 10^−1^	3.0–9.0	30.0 µVs^−1^ (i ± 1 nA)	[174]
Ca^2+^	calcium ionophore II	EDOT-S	28.9	4.5 × 10^−7^	1.0 × 10^−6^–1.0 × 10^−1^	-	300 μVh^–1^(i = 0)	[161]
Ca^2+^	4,7-diaza-2,3,8,9-dibenzo-15- crown-5	MWCNT in PVC	28.8	9.1 × 10^−8^	1.6 × 10^−7^–1.0 × 10^−1^	3.5–7.0	-	[143]
Cu^2+^	copper(II) ionophore IV	SBS-BMImPF_6_	28.8	-	1.0 × 10^−6^–1.0 × 10^−1^	-	-	[148]
Cu^2+^	copper(II) ionophore IV	ETH 500/SWCNTs ETH 500/graphene	-	4.0 × 10^−9^	1.0 × 10^−8^–1.0 × 10^−4^	-	-	[164]
Cu^2+^	copper(II) ionophore IV	graphene/TCNQ,TCNQ-Cu nanocomposite	29.9	2.5 × 10^−9^	1.0 × 10^−8^–1.0 × 10^−2^	4.0–6.0	25.2 µVs^−1^ (i = ± 10 nA)	[167]
Cu^2+^	copper(II)-ion-imprinted polymer–graphite oxide nanocomposite		26.1	4.0 × 10^−7^	1.0 × 10^−6^–1.0 × 10^−1^	4.0–8.0	-	[139]
Cu^2+^	copper(II) ionophore IV	MWCNTs:BMImPF_6_	29.8	3.3 × 10^−8^	1.0 × 10^−7^ –1.0 × 10^−2^	2.5– 6.0	2760 µVh^−1^ (i = 0)	[170]
Cu^2+^	copper(II) ionophore IV	CuONPs-MWCNTs nanocomposite	30.1	1.5 × 10^−8^	5.0 × 10^−8^–3.0 × 10^−2^	4.0–6.0	132.0 µV h^−1^ (i = 0)	[179]
Cd^2+^	cadmium ionophore I	3D PGR-MPN	29.6	1.6 × 10^−.^	1.0 × 10^−8^–3.0 × 10^−4^	-	1.6 µVs^−1^ (i ± 1 nA)	[168]
Ce^3+^	benzo-15-crown-5	CoNiFe_2_O_4_ nanocomposite	17.5	7.0 × 10^−9^	1.0 × 10^−8^–1.0 × 10^−1^	2.0–10.0	-	[185]
Fe^2+^	MWCNTs-Gemi composite	graphite powder	30.4	4.8 × 10^−9^	1.0 × 10^−8^–1.0 × 10^−2^	3.0–8.0	-	[172]
Pb^2+^	lead ionophore IV	SBS-BMImPF_6_	28.3	–	1.0 × 10^−6^–1.0 × 10^−2^	-	−	[148]
Pb^2+^	lead ionophore IV	ETH 500/SWCNTs ETH 500/graphene	-	1.8 × 10^−9^	1.0 × 10^−9^–1.0 × 10^−4^	-	-	[164]
Pb^2+^	lead ionophore IV	MWCNTs	29.0	4.0 × 10^−10^	2.0 × 10^−3^–2.0 × 10^−9^	2.0–4.8	19.8 µVs^−1^ (i = ± 1 nA)	[141]
Pb^2+^	lead ionophore IV	e-PANI-PS	29.1	5.0 × 10^−9^	1.0 × 10^−8^–1.0 × 10^−3^	-	12.3 µVs^−1^ (i = ± 1 nA)	[154]
Pb^2+^	lead ionophore IV	PANI-TiO_2_	29.0	7.9 × 10^−10^	1.0 × 10^−9^–1.0 × 10^−3^	-	122.6 µVs^−1^ (i = ± 1 nA)	[156]
Pb^2+^	lead ionophore IV	Ag@PANI	29.1	6.3 × 10^−10^	1.0 × 10^−9^–1.0 × 10^−3^	3.0–9.0	25.1 µVs^−1^ (i ± 1 nA)	[159]
Pb^2+^	lead ionophore IV	CNFs:HMImPF_6_	31.5	6.0 × 10^−9^	1.0 × 10^−8^–1.0 × 10^−2^	3.1–7.6	105 µVh^−1^ (i = 0)	[178]
NO_3_^−^	TDMANO_3_	PtNPs-CB	−58.6	5.0 × 10^−7^	1.0 × 10^−6^–1.0 × 10^−1^	3.0–9.0	6.3 µVh^−1^ (i = 0)	[163]
NO_3_^−^	nitrate ionophore V	graphene-TTF/TTF^+^	−59.1	6.3 × 10^−7^	1.0 × 10^−6^–1.0 × 10^−1^	-	4.26 µVs^−1^ (i = ± 5 nA)	[166]
NO_3_^−^	nitrate ionophore V	TTF-TCNQ	−58.5	3.2 × 10^−6^	1.0 × 10^−5^–1.0 × 10^−1^	-	16.7 µVs^−1^ (i = ± 10 nA)	[182]
NO_3_^−^	TDMACl	MOFs	−56.3	6.3 × 10^−7^	1.0 × 10^−6^–3.0 × 10^−2^	-	11.1 µVh^−1^ (i = 0)	[183]
NO_3_^−^	TDMANO_3_	POT:MoS_2_ nanocomposite	−64.0	9.2 × 10^−5^	7.1 × 10^−4^–1.0 × 10^−1^	-	-	[155]
NO_3_^−^	nitrate ionophore V	PAAm(Cl^−^)-MnO_2_	−50.6	6.3 × 10^−6^	6.3 × 10^−6^–1.0 × 10^−1^	-	2.0 µVs^−1^ (i ± 1 nA)	[160]
NO_3_^−^	Co(Bphen)_2_(NO_3_)_2_	MWCNTs:THTDPCl nanocomposite	−57.1	5.0 × 10^−7^	1.0 × 10^−6^–1.0 × 10^−1^	4.2–10.8	106 μVs^−1^ (i = ± 100 nA) 151.2 µVh^−1^ (i = 0)	[169]
NO_3_^−^	TDDANO_3_	PEDOT:PEG	−55.8	1.1 × 10^−6^	1.1 × 10^−6^–1.0 × 10^−1^	4.0–10.0	280 μVh^−1^ (i = 0), 90.9 μVs^−1^ (i = ± 10 nA)	[162]
Cl^−^	TDMACl	CB-TTF-TCNQ	−58.7	2.5 × 10^−6^	1.0 × 10^−5^–1.0 × 10^−1^	-	16.0 µVs^−1^ (i = ± 300 nA)	[152]
Cl^−^	bisthiourea-1	PAAm(Cl^−^)-MnO_2_	−52.2	1.0 × 10^−5^	1.0 × 10^−5^–1.0 × 10^−1^	–	3300 μVs^−1^ (i ± 1 nA)	[160]
Cl^−^	chloride ionophore III	PANINFs-Cl:MWCNT nanocomposite	−61.3	2.3 × 10^−6^	5.0 × 10^−6^–1.0 × 10^−1^	4.0–9.0	30 μVh^−1^ (i = 0)	[144]
SO_4_^2−^	sulphate ionophore I	oAuCuNPs-MWCNTs	−27.0	9.5 × 10^−6^	1.0 × 10^−5^–1.0 × 10^−1^	-	7080 µVh^−1^ (i = 0)	[119]
H_2_PO_4_^−^	uranyl salophene ionophore I	MWCNTs-F127 nanocomposite	−59.0	1.6 × 10^−5^	3.2 × 10^−5^–1.0 × 10^−1^	-	-	[165]

**Alphabetical list of abbreviations (to Table 1):** 3D graphene-CNT—3D graphene oxide–carbon nanotubes composite; 3D PGR-MPN—three-dimensional porous graphene–mesoporous platinum nanoparticles composite; Ag@PANI—silver nanoparticles and polyaniline; ammonium ionophore I—nonactin; Au/CNT/Au—gold–carbon nanotube–gold sensors; AU-1—N,N-dicyclohexyl-N’-3-(2-propenoyl)-oxyphenyl-3-oxapentanediamide; bisthiourea-1—N-(butyl)thioureido-2,7-di-tert-butyl-9,9-dimethylxanthene; cadmium ionophore I—N,N,N’,N’-tetrabutyl-3,6-dioxaoctanedi(thioamide); calcium ionophore II—2-[2-(dicyclohexylamino)-2-oxoethoxy]-N,N-dio-ctadecylacetamide, ETH 5234; CA—composite aerogel of cellulose fibers and carbon nanotubes; CB-FP—the highly porous graphene/carbon black fluorinated acrylic copolymer; chloride ionophore III—3,6-didodecyloxy-4,5-dimethyl-o-phenylene-bis(mercury chloride), ETH 9033; CB/POT/hIrO_2—_carbon black/poly(3-octylthiophene-2,5-diyl)/hydrous iridium dioxide triple composite; ChPBN nanocomposite—solution-casted chitosan/Prussian blue nanocomposite; CNFs:HMImPF_6_—carbon nanofibers with 1-hexyl-3-methylimidazolium hexafluorophosphate nanocomposite; CNT/POT/hIrO_2_—carbon nanotubes/poly(3-octylthiophene-2,5-diyl)/hydrous iridium dioxide triple composite; CNT-PVC—composite polyvinyl chloride membrane impregnated with carbon nanotubes; Co(Bphen)_2_(NO_3_)_2_—cobalt(II) complex with 4,7-diphenyl-1,10-phenanthroline; CoNiFe_2_O_4_ nanocomposite—cobalt-doped nickel iron oxide nanocomposite; copper(II) ionophore IV—N,N,N′,N′-tetracyclohexyl-2,2′-thiodiacetamide; CPANI—copolymer of aniline/2,5-dimethoxyaniline; CuONPs-MWCNTs nanocomposite—copper(II) oxide nanoparticles and multiwalled carbon nanotubes nanocomposite; eCNF, eCNF-Co and eCNF/CNT-NiCo with POT nanocomposites—electrospun carbon nanofibers, electrospun carbon nanofibers with embedded cobalt nanoparticles and hierarchical nanocomposite with the nanoparticles of cobalt and nickel as a catalyst for the growth of carbon nanotubes with poly(3-octylthiophene-2,5-diyl); eCNF/CNT[HD]-NiCo—nanocomposite of electrospun carbon nanofibers and carbon nanotubes with NiCo nanoparticles as growth catalysts differing in the surface density of CNTs, here HD—high density; EDOT-S—3,4-ethylenedioxythiophene with 4-(2,3-dihydrothieno [3,4-b][1,4]dioxin-2-yl-methoxy)-1-butanesulfonic acid, sodium salt; e-PANI-PS—hydrophobic polyaniline-polystyrene microfiber films; ETH 500/graphene—tetradodecylammoniumtetrakis(4-chlorophenyl)borate/graphene mixture; ETH 500/SWCNTs—tetradodecylammoniumtetrakis(4-chlorophenyl)borate/single-walled carbon nanotubes mixture; GR + RuO_2_, CB + RuO_2_, NT + RuO_2_—nanocomposites of single-layer graphene, carbon black and multiwalled carbon nanotubes with ruthenium dioxide nanoparticles; graphene/TCNQ, TCNQ-Cu nanocomposite—graphene and 7,7,8,8-tetracyanoquinodimethane nanocomposite; graphene-TTF/TTF^+^—graphene/tetrathiafulvalene nanocomposite; graphite-PVB—graphite particles embedded in a polyvinyl butyral matrix; hCeO_2_, hCeO_2_ + NTs, hCeO_2_ + POT—hydrous cerium oxide, hydrous cerium oxide-carbon nanotubes, hydrous cerium oxide-conducting polymer composite materials; HMInPF_6_—1-hexyl-3-methylimidazolium hexafluorophosphate; lariat ether—7-[(2-hydroxy-1-naphthyl)methyl]-5,6,7,8,9,10-hexahydro-2H-1,13,4,7,10-benzodioxatriazacyclopentadecine-3,11(4H,12H)-dione; lead ionophore IV—tert-butylcalix [4]arene-tetrakis(N,N-dimethylthioacetamide); lithium ionophore VI—6,6-dibenzyl-1,4,8-11-tetraoxacyclotetradecane; MMA–DMA copolymer—plasticizer-free methyl methacrylate–decyl methacrylate copolymer; MMWCNTs@Cs-IIP—magnetic multiwalled carbon nanotubes/cesium-ion-imprinted polymer composite; MOFs—conductive metal-organic frameworks; MoS_2_/Fe_3_O_4_—MoS_2_ nanoflowers with Fe_3_O_4_ nanoparticles nanocomposite; MWCNTs:BMImPF_6_—multiwalled carbon nanotubes with 1-butyl-3-methylimidazolium hexafluorophosphate; MWCNTs:THTDPCl nanocomposite—multiwalled carbon nanotubes and trihexyltetradecylphosphonium chloride nanocomposite; MWCNTs-F127—pluronic F-127 and multiwalled carbon nanotubes nanocomposite; MWCNTs-Gemi composite—multiwalled carbon nanotubes with gemifloxacin composite; Na(X)—4-tert-butylcalix [4]arene-tetraacetic acid tetraethyl ester; nitrate ionophore V—5,7,16,18-tetrahydrotetrabenzo[d,f,k,m][1,3,8,10]tetraazacyclotetradecine-6,17-dithione; oAuCuNPs-MWCNTs—ordered bimetallic AuCu nanoparticles coupled with multiwalled carbon nanotubes; PAAm(Cl^−^)-MnO_2_—poly(allylamine) and manganese dioxide composite; PANI-graphene—polyaniline nanofibers and graphene composite; PANINFs-Cl:MWCNTs nanocomposite—chloride-doped polyaniline nanofibers and multiwalled carbon nanotubes nanocomposite; PANI-ZrI—polyaniline-zirconium(IV) iodate; Pd/rGO/PDA@NF—composite material of spherical palladium nanoparticles, reduced graphene oxide and polydopamine on three-dimensional nickel foam; PEDOT(CNT)—poly(3,4-ethylenedioxythiophene) doped with carbon nanotubes composite; PEDOT(PSS)—poly(3,4-ethylenedioxythiophene) doped with poly(sodium 4-styrenesulfonate); PEDOT:PEG—poly(3,4-ethylenedioxythiophene)-polyethylene glycol; PEDOT-HQ—conjugated redox polymer with hydroquinone pendant groups covalently attached to the poly(3,4-ethylenedioxythiophene); PMMA-CeMoO_4_—poly(methyl methacrylate)-cerium molybdate nanocomposite; POT:MoS_2_—poly(3-octyl-thiophene) and molybdenum disulfide nanocomposite; potassium ionophore I—valinomycin; potassium ionophore II—bis[(benzo-15-crown-5)-15-ylmethyl]pimelate; POT-MWCNTs—poly(3-octylthiophene-2,5-diyl) and multiwalled carbon nanotubes nanocomposite; PPy/H-ZSM-5—electrosynthesized polypyrrole/zeolite composites; PtNPs-CB—carbon black supporting platinum nanoparticles; PtNPs-GR—graphene supporting platinum nanoparticles; RuO_2_ + POT—ruthenium dioxide and poly(3-octylthiophene-2,5-diyl) composite material; RuO_2_-PEDOT:PSS—ruthenium dioxide–poly(3,4-ethylenedioxythiophene) polystyrene sulfonate nanocomposite; sodium ionophore VI—bis[(12-crown-4)methyl] dodecylmethylmalonate, dodecylmethylmalonic acid bis[(12-crown-4)methyl ester]; sodium ionophore X—4-tert-butylcalix [4-arene-tetraacetic acid tetraethyl ester]; sulfate ionophore I—α,α’-bis(N’-phenylthioureylene)-m-xylene, bisthiourea; TDDANO_3_—tetradodecylammonium nitrate; TDMACl—tridodecylmethylammonium chloride; TDMANO_3_—tridodecylmethylammonium nitrate; TPEs—thermoplastic electrodes, polystyrene-graphite nanoplatelets; TTF-TCNQ—tetrathiafulvalene-tetracyanoquinodimethane.

## Data Availability

Not applicable.
